# Generation and evaluation of antibody agents for molecular imaging of CD44v6-expressing cancers

**DOI:** 10.18632/oncotarget.17996

**Published:** 2017-05-18

**Authors:** Anna-Karin Haylock, Johan Nilvebrant, Anja Mortensen, Irina Velikyan, Marika Nestor, Ronny Falk

**Affiliations:** ^1^ Department of Surgical Sciences, Uppsala University, Uppsala, Sweden; ^2^ Department of Immunology, Genetics and Pathology, Uppsala University, Uppsala, Sweden; ^3^ Division of Protein Technology, School of Biotechnology, Royal Institute of Technology, Stockholm, Sweden; ^4^ Department of Medicinal Chemistry, Uppsala University, Uppsala, Sweden; ^5^ Department of Neuroscience, Karolinska Institutet, Stockholm, Sweden

**Keywords:** scFv, recombinant antibody formats, CD44v6, squamous cell carcinoma, molecular imaging

## Abstract

**Aim:**

The aim of this study was to generate and characterize scFv antibodies directed to human CD44v6, as well as to radiolabel and evaluate top candidates *in vitro* and *in vivo* for their potential use in CD44v6-targeted molecular imaging in cancer patients.

**Materials and methods:**

Phage display selections were used to isolate CD44v6-specific scFvs. A chain shuffling strategy was employed for affinity maturation based on a set of CD44v6-specific first-generation clones. Two second-generation scFv clones were then chosen for labeling with ^111^In or ^125^I and assessed for CD44v6-specific binding on cultured tumor cells. *In vivo* uptake and distribution was evaluated in tumor-bearing mice using a dual tumor model. Finally, a proof-of-concept small animal PET-CT study was performed on one of the candidates labeled with ^124^I.

**Results:**

Two affinity-matured clones, CD44v6-scFv-A11 and CD44v6-scFv-H12, displayed promising binding kinetics. Seven out of eight radiolabeled conjugates demonstrated CD44v6-specific binding. *In vivo* studies on selected candidates demonstrated very advantageous tumor-to-organ ratios, in particular for iodinated conjugates, where ^125^I-labeled scFvs exhibited favorable kinetics and tumor-to-blood ratios above five already at 24 hours p.i.. The small animal PET-CT study using ^124^I-labeled CD44v6-scFv-H12 was in line with the biodistribution data, clearly visualizing the high CD44v6-expressing tumor.

**Conclusion:**

The single chain fragments, CD44v6-scFv-A11 and CD44v6-scFv-H12 specifically bind to CD44v6, and the radiolabeled counterparts provide high tumor-to-blood ratios and fast clearance from organs and blood. We conclude that radioiodinated CD44v6-scFv-A11 and CD44v6-scFv-H12 possess features highly suitable for stringent molecular imaging.

## INTRODUCTION

The development of molecular imaging modalities such as positron emission tomography (PET) and single photon emission computed tomography (SPECT) continues to progress, owing to the ability of these techniques to allow non-invasive *in vivo* visualization of biological processes at the molecular and cellular levels. Crucial to the growth of these techniques is the continued development of radionuclide labeled targeting agents that can bind to the target receptor with high selectivity. In head and neck squamous cell carcinoma (HNSCC), 2-deoxy-2-[^18^F]-fluoroglucose ([^18^F]FDG-PET) is widely used as a diagnostic tool, and to monitor treatment response after therapy. However, the positive predictive value of this technique post treatment is only around 50% in patients [1, 2], mainly due to factors such as the high occurrence of post treatment inflammatory reactions [3]. Consequently, a method that could specifically detect occult metastases, residual or recurrent disease at an earlier stage post treatment hold promise to improve prognosis [4].

Antibody-based radionuclide imaging is an attractive tool for diagnostics of HNSCC. Such imaging agents combine the imaging ability of PET and SPECT with tumor specificity. However, intact monoclonal antibodies (mAbs) are large (150 kDa) molecules, with generally slow pharmacokinetics, slow blood clearance, and sub optimal tumor penetration and accumulation [5-8]. Antibody fragments such as single chain fragment variable (scFv) have shown great promise in imaging applications [9] including cancer diagnostics, where the scFv format has been shown to provide a good balance of system clearance, tumor penetration and tissue accumulation [7, 10-12]. Traditionally, antibody production involves immunization to generate polyclonal antibody preparations and, for production of monoclonal antibodies, immunization combined with the hybridoma technology [13]. More recently, selection of antibody fragments using display technologies offers an *in vitro* strategy to isolate antibody fragments that can be produced recombinantly in bacterial hosts [14, 15]. With *in vitro* antibody selection it is possible to precisely control the selection conditions to promote generation of binders with sought properties. Furthermore, recombinant antibody formats provide immediate availability of recovered antibody genes, which facilitates downstream optimization [12, 16].

Several studies have demonstrated overexpression of CD44v6 in over 90% of primary and metastatic HNSCC [17, 18], and it has been suggested to play a role in tumor formation, invasion, and metastasis formation [17, 19]. The CD44 gene is made up of 20 exons among which 10 exons are variable. Alternative expression generates CD44 variants (CD44v) (Supplementary Figure 1). These isoforms can in turn undergo post- translational modifications [20]. CD44v6 expression in normal tissues is restricted to some squamous and transitional epithelia [21, 22]. Thus, CD44v6 is a promising candidate antigen for targeting of squamous cell carcinoma [23]. Previous clinical studies with HNSCC targeting CD44v6 with chimeric and humanized monoclonal antibodies, including the antibody U36, have shown potential for antibody based molecular imaging [24-26].

Previous studies with labeled CD44v6-targeting Fab and F(ab’)_2_ have shown favorable results regarding tumor contrast, albeit with room for improvement in tumor uptake and penetration, and blood clearance [27-29]. A smaller agent has potential to produce faster blood clearance and enhanced tumor penetration, and consequently higher contrast, thereby enabling imaging at an earlier time point post injection. Thus, the aim of this study was to select and evaluate novel CD44v6-targeting scFv fragments, and to generate and assess suitable radiolabeled analogues from the top candidates *in vitro* and *in vivo* for potential use as CD44v6-targeting molecular imaging agents.

## RESULTS

### Antibody selection and affinity maturation

**Figure 1:**
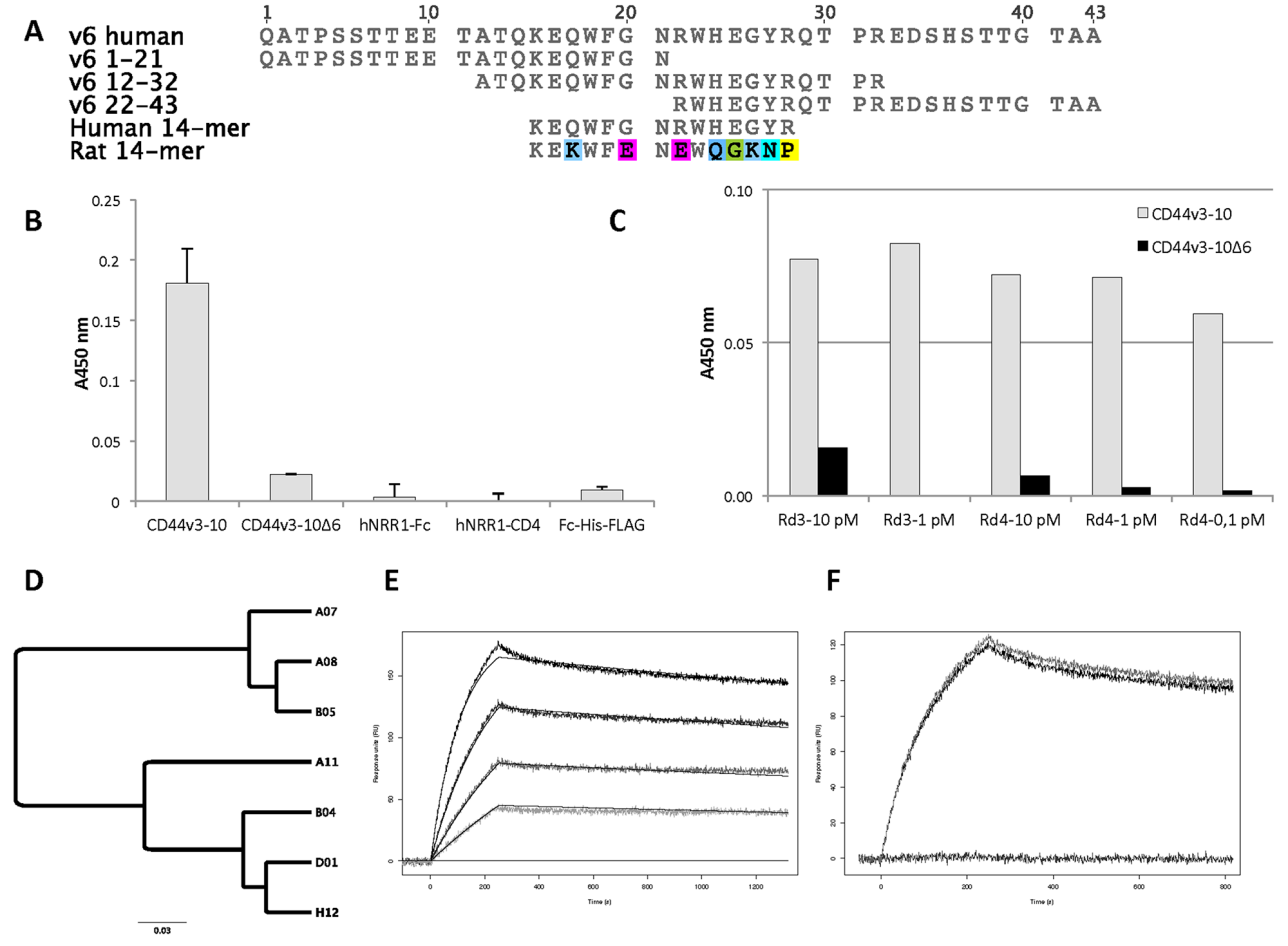
Antigens, post-selection analysis and characterization of scFv clones **(A)** Sequence alignment of v6 region of human CD44v6 and peptides used in this study. v6 1-21, v6 12-32 and v6 22-43 are overlapping peptides that span the entire sequence of v6. 14-mer peptides derived from human and rat CD44v6, respectively, were used for blocking and species specificity testing. **(B)** Phage-scFv ELISA with the population isolated after two selection rounds. Binding is specific to the v6-positive antigen, CD44v3-10, with very limited binding to the v6-negative protein, CD44v3-10Δv6, and no binding to three other proteins (hNRR1-Fc, hNRR1-CD4 and Fc-His-FLAG) included as controls. Bars and standard deviation are from two reads of each sample. Background signal from a negative control (no phage) is subtracted. **(C)** ELISA with polyclonal phage populations from affinity maturation selection rounds 3 (Rd3) and 4 (Rd4). Antigen concentrations used to select respective populations were between 10 - 0.1 pM as indicated. All populations have specific binding to CD44v3-10 (grey) with very limited reactivity to CD44v3-10Δv6 (black). A450 from background (no phage) has been subtracted. **(D)** Cluster of selected affinity-matured scFv clones. Seven clones identified after affinity maturation are included, the top candidates scFv-A11 and scFv-H12 (indicated in bold) belong to the same high affinity cluster. The scale bar indicates the number of substitutions per residue, i.e. a longer distance corresponds to larger differences between the sequences. The sequence similarity tree was constructed using the Geneious software v7.1.9 (Biomatters, Auckland, New Zealand). **(E)** Representative sensorgram for scFv-A11 binding to immobilized CD44v3-10. The curves represent 50, 25, 12.5 and 6.3 nM, respectively. Solid lines are fitted to a 1:1 Langmuir binding isotherm using the ProteOn Manager software 3.1.0.6. **(F)** Blocking and species specificity of scFv-A11. Both scFv-A11 alone (50 nM) and scFv-A11 pre-incubated with a 10-fold molar excess of rat 14-mer peptide bind to immobilized CD44v3-10 (top two curves). Pre-incubation with human 14-mer peptide blocked binding (lower curve). Injection of 14-mers alone did not give any signal (not shown).

To generate antibodies specific for CD44v6 we selected binders targeting the v6-region of the antigen (Figure 1A) from a scFv phage display library. Binding specificity of the isolated scFv-phage population was evaluated in ELISA after two selection rounds. The polyclonal pool displayed specific binding to CD44v6 with very low binding to CD44v3-10Δv6, a protein representing regions shared with other isoforms of CD44 that lacks the v6 region, and three other proteins (hNRR1-Fc, hNRR1-CD4 and Fc-His-FLAG) included as negative controls (Figure 1B). The low reactivity to CD44v3-10Δv6 suggests that the initial de-selection step against this protein efficiently depleted antibodies binding to common residues flanking the v6 region of CD44v6.

To identify individual clones the selected population was subcloned for soluble expression in BL21(DE3) cells. From an ELISA screen of 384 clones (Supplementary Figure 1) the 96 clones with highest signal were chosen for DNA-sequencing, which identified 25 unique scFv genes (data not shown). The sequence unique clones were tested for binding to endogenous CD44v6 on A431 cells in flow cytrometric analysis. A majority of the clones showed limited binding to A431 cells. However, due to low and varying antibody concentrations, these data were not considered when candidates for expression and characterization were selected (data not shown).

A subset of clones, representing different clusters of scFv with high similarity, was selected for a binding analysis by SPR to determine their binding properties. Although only weak binding to immobilised protein could be observed (data not shown), the results indicated specific binding to CD44v6. This was in agreement with our previous ELISA results (Figure 1A and Supplementary Figure 1).

To generate scFv variants with improved affinity to increase the utility of single chain antibodies in subsequent *in vivo* applications, we recombined V_H_ chains from the 25 clones specifically binding CD44v6 with a naive V_L_ repertoire and achieved a, chain shuffled, CD44v6-scFv library. From this library, matured clones were successfully selected by an initial biopanning round followed by three additional rounds involving incubation with low concentrations of biotinylated CD44v3-10 in solution. The scFv-phage populations isolated from the affinity maturation selections were analyzed in a polyclonal phage ELISA, and specific binding to CD44v3-10, with only weak cross reactivity to CD44v3-10Δv6, could be verified for phage populations rescued after rounds 3 and 4 (Figure 1C). Populations from the last two rounds were chosen for subcloning and soluble expression of individual scFv clones to screen for clones that bound CD44v3-10 in ELISA. From 384 screened clones the 96 scFv clones generating the highest ELISA signals (data not shown) were chosen for DNA sequencing and 14 clones with unique sequence were identified. Based on sequence similarity, a subset of second-generation scFv clones representing two dominating clusters of scFv sequences (Figure 1D) were selected for further analysis.

### Surface plasmon resonance

Binding affinity for seven selected affinity-matured scFv clones was determined by SPR (Table 1). The clones with highest affinities demonstrated subnanomolar dissociation equilibrium constants (K_D_), a representative sensorgram for CD44v6-scFv-A11 is shown in Figure 1E. All clones bound CD44v3-10 with no observed cross-reactivity on a v6-negative CD44 isoform (CD44v3-10Δv6). Moreover, all clones bound v6 peptide 12-32 with no signals on peptides 1-21 or 22-43 (data not shown, see Figure 1A for peptide sequences). Hence, the binding epitope could be mapped to residues 12-32 of CD44v6. Pre-incubation of scFv with an excess of a human 14-mer peptide, corresponding to the central part of the 12-32 peptide (Figure 1A), prior to injection effectively blocked binding to immobilized CD44v3-10 (Figure 1F), which indicated that the epitope is located within residues KEQWFGNRWHEGYR of CD44v6. Lack of competition with an equivalent 14-mer derived from rat CD44v6 suggests that binding was specific for human CD44v6 (Figure 1F).

**Table 1:** Binding kinetics of CD44v6-binding scFvs. Average association- and dissociation rate constants from the indicated number of replicate measurements are shown with standard deviations. Dissociation equilibrium constants were calculated as k_d_/k_a_

Candidate	k_a_ (M^-1^s^-1^)	k_d_ (s^-1^)	K_D_ (nM)	Number of replicates
CD44v6-scFv-A7	2.1(±0.7)*10^5^	5.7(±0.9)*10^-3^	27	4
CD44v6-scFv-A8	0.9(±0.1)*10^5^	3.3(±0.2)*10^-3^	37	4
CD44v6-scFv-A11	2.1(±0.6)*10^5^	1.7(±1.0)*10^-4^	0.8	8
CD44v6-scFv-B4	3.7(±1.1)*10^5^	3.8(±1.1)*10^-4^	1.0	6
CD44v6-scFv-B5	0.5(±0.1)*10^5^	3.5(±0.4)*10^-3^	70	2
CD44v6-scFv-D1	5.1(±2.4)*10^5^	9.9(±1.9)*10^-4^	1.9	6
CD44v6-scFv-H12	5.5(±1.1)*10^5^	2.7(±0.7)*10^-4^	0.5	6

### Labeling

Iodination using CAT or Iodogen resulted in radiochemical yields of 70 – 90% and 36 - 51%, respectively. Labeling with ^111^In using DOTA or CHX-A”-DTPA resulted in radiochemical yields of 15 – 20% and 95 – 100% (non-decay corrected), respectively. Radiochemical purity was > 95% already in the reaction vial for ^111^In-DTPA-A11 and ^111^In-DTPA-H12, hence no size-exclusion step was performed on this solution. For all other conjugates, the radiochemical purity of selected fractions after size-exclusion chromatography purification was > 95% and was unchanged after storage in PBS for 24 h according to ITLC analysis. Stability for ^111^In labeled conjugates was also assessed by EDTA challenge. Thirty minutes of incubation in 500-fold molar excess of EDTA at 37°C did not significantly alter the radiochemical purity of ^111^In-DTPA-A11 (97.9%) and ^111^In-DOTA-A11 (93.8%). For ^111^In-DTPA-H12 and ^111^In-DOTA-H12 however, the radiochemical purity was decreased by EDTA challenge from > 95%, respectively to 86.3% and 68.7% after 30 min and to 69.5% and 67.1% after 24 h.

For radioiodinations, specific activities were around 260 kBq/µg and 180 kBq/µg for ^125^I-A11(Iodogen) and ^125^I-H12(Iodogen) respectively, and approximately 270 kBq/µg and 230 kBq/µg for ^125^I-A11(CAT) and ^125^I-H12(CAT) respectively. Specific activities of the ^111^In-labeled analogues used in *in vitro* experiments were typically around 260 kBq/µg and 280 kBq/µg for ^111^In-DOTA-A11 and ^111^In-DOTA-H12, respectively, and around 400 kBq/µg and 440 kBq/µg for ^111^In-DTPA-A11 and ^111^In-DTPA-H12, respectively. For animal studies, specific activities were adjusted to 78 kBq/µg and 58 kBq/µg for ^125^I-A11(Iodogen) and ^125^I-H12(Iodogen), respectively, and to 167 kBq/µg for both ^111^In-DTPA-A11 and ^111^In-DTPA-H12 conjugates. For the small animal PET-CT study, specific activity of ^124^I-H12(CAT) was 700 kBq/µg.

### Real time binding comparisons of labeled conjugates

**Figure 2:**
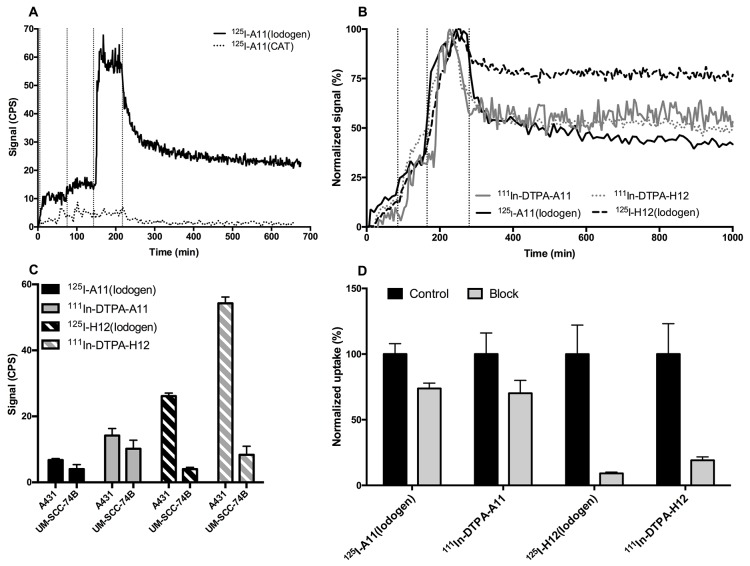
*In vitro* cell binding of radiolabeled conjugates **(A)** Representative LigandTracer measurements of real-time binding of CD44v6-scFv-A11 labeled with 125I using either Iodogen (top curve) or Chloramine T (bottom curve, dotted line) on A431 cells. Binding traces using three subsequent concentrations (10, 20, 60 nM) were obtained for at least 1 h per concentration (marked by vertical dotted lines), followed by a dissociation measurement for at least 10 h. CPS = Counts per second. **(B)** Representative LigandTracer measurements of real-time binding of CD44v6-scFv-A11 (filled lines) and CD44v6-scFv-H12 (dotted lines) on A431 cells, normalized to percentage of maximum binding. Binders were labeled with either ^111^In (grey lines) or ^125^I (black lines). Binding traces using three subsequent concentrations (1, 3, 9 nM) were obtained for at least 1 h per concentration (marked by vertical dotted lines), followed by a dissociation measurement for at least 10 h. **(C)** Signal intensities during equilibrated conditions after 2 h of incubation with 9 nM of conjugate using LigandTracer. Incubation was preceded by 2 + 2 h of uptake measurements at 1 and 3 nM. Measurements were performed using ^125^I-A11(Iodogen) (52.5 ng, 4 kBq, black bars), ^125^I-H12(Iodogen) (52.5 ng, 3 kBq, black striped bars), ^111^In-DTPA-A11 (52.5 ng, 8.8 kBq, grey bars) and ^111^In-DTPA-H12 (52.5 ng, 8.8 kBq, grey striped bars) on A431 cells of high CD44v6 density and UM-SCC-74B cells of low CD44v6 density. Signal from target area (cells) were subtracted by signal from the background area. CPS = Counts per second. Error bars show standard deviation (N=5). **(D)** Blocking assays for 125I-A11(Iodogen), 125I-H12(Iodogen), 111In-DTPA-A11 and 111In-DTPA-H12 on A431 cells. Radiolabeled conjugate was added at a concentration of 10 nM. In blocked samples, 100-times molar excess of non-labeled binder was also added to block specific binding of labeled conjugate. The samples were allowed to incubate for 4 hours at 37 °C, 5% CO2. Differences between unblocked and blocked samples were found to be significant for all conjugates according to student’s t test (P<0.05). Error bars show standard deviation (N>5).

The binding of the radiolabeled scFv to CD44v6-expressing cells was first compared using LigandTracer on high CD44v6-expressing A431 cells. For iodinated CD44v6-scFv-A11 tracers, ^125^I-A11(Iodogen) and ^125^I-A11(CAT) demonstrated distinctly different results, where ^125^I-A11(Iodogen) displayed clear target binding and ^125^I-A11(CAT) did not (Figure 2A). For CD44v6-scFv-H12 however, the iodinated tracers ^125^I-H12(Iodogen), ^125^I-H12(CAT) and ^124^I-H12(CAT) demonstrated matching binding traces. Furthermore, LigandTracer measurements of ^111^In-DOTA-A11, ^111^In-DOTA-H12, ^111^In-DTPA-A11, and ^111^In-DTPA-H12 all resulted in similar binding patterns on A431 cells with minor differences between conjugates (data not shown).

^125^I labeling using Iodogen, and ^111^In labeling using CHX-A”-DTPA was chosen for further evaluations of real-time binding in both low CD44v6-expressing UM-SCC-74B cells and high CD44v6-expressing A413 cells (Figure 2B and 2C). As seen in Figure 2B, the conjugates demonstrated similar on-rates on A431 cells, ranging between 1.5*10^4^ and 19.8*10^4^ M^−1^ s^−1^ for all measurements. Also retention on A431 cells was comparable between conjugates, with the exception for ^125^I-H12(Iodogen), where a larger fraction remained bound to the cells during retention phase. However, after the initial drop in target binding after start of retention measurements (approximately 20 min), all conjugates displayed very slow off-rates of 1.5*10^-5^ s^−1^ or lower (Figure 2B). As expected, the highest target signals were obtained on the high CD44v6-expressing A431 cells for all conjugates, whereas signals barely above the detection limit were obtained on the low CD44v6-expressing UM-SCC-74B cells (Figure 2C), demonstrating the specificity of the binders.^125^I-H12 (Iodogen) demonstrated a higher target signal than ^125^I-A11(Iodogen), and ^111^In-DTPA-H12 demonstrated a higher target signal than ^111^In-DTPA-A11 (Figure 2C).

### Blocking assays

Blocking assays were performed on ^125^I-A11(Iodogen), ^125^I-H12(Iodogen), ^111^In-DTPA-A11 and ^111^In-DTPA-H12. Binding of labeled scFv was significantly decreased by an excess of unlabeled binder for all conjugates (Figure 2D). As observed in the LigandTracer assays, the obtained binding to cellular CD44v6 on unblocked samples were superior for ^125^I-H12(Iodogen) compared to ^125^I-A11(Iodogen), and for ^111^In-DTPA-H12 compared to ^111^In-DTPA-A11, displaying four and six times higher binding signals for the ^111^In-DTPA-H12 and ^125^I-H12(Iodogen) conjugates, respectively. Consequently, the signal intensities of the ^125^I-H12(Iodogen) and ^111^In-DTPA-H12 were decreased to 9 ± 1 (SEM)% and 19 ± 3% respectively in the presence of non-labeled CD44v6-scFv-H12 excess as compared to unblocked samples. The respective values for ^125^I-A11(Iodogen) and ^111^In-DTPA-A11 were 74 ± 4% and 79 ± 5%.

### Ex vivo immunohistochemistry

**Figure 3:**
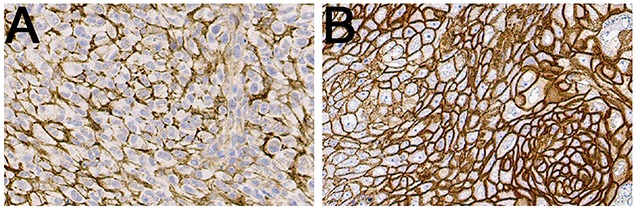
Representative IHC images of CD44v6 stainings of UM-SCC-74B (**A**) and A431 (**B**) xenografted tumors.

To assess degree of CD44v6 expression in the tumors in the grafted animals, IHC analyses with an additional antibody specific to CD44v6 was conducted. Representative images of *ex vivo* immunohistochemistry stainings can be seen in Figure 3. Both A431 tumors and UM-SCC-74B tumors contained viable tumor and stromal cells, well established blood vessels and minor areas of necrosis. A431 tumors displayed a very strong staining of CD44v6 whereas a lower, albeit clear, CD44v6-staining was seen in UM-SCC-74B tumors.

### *In vivo* target binding in small animals

**Figure 4:**
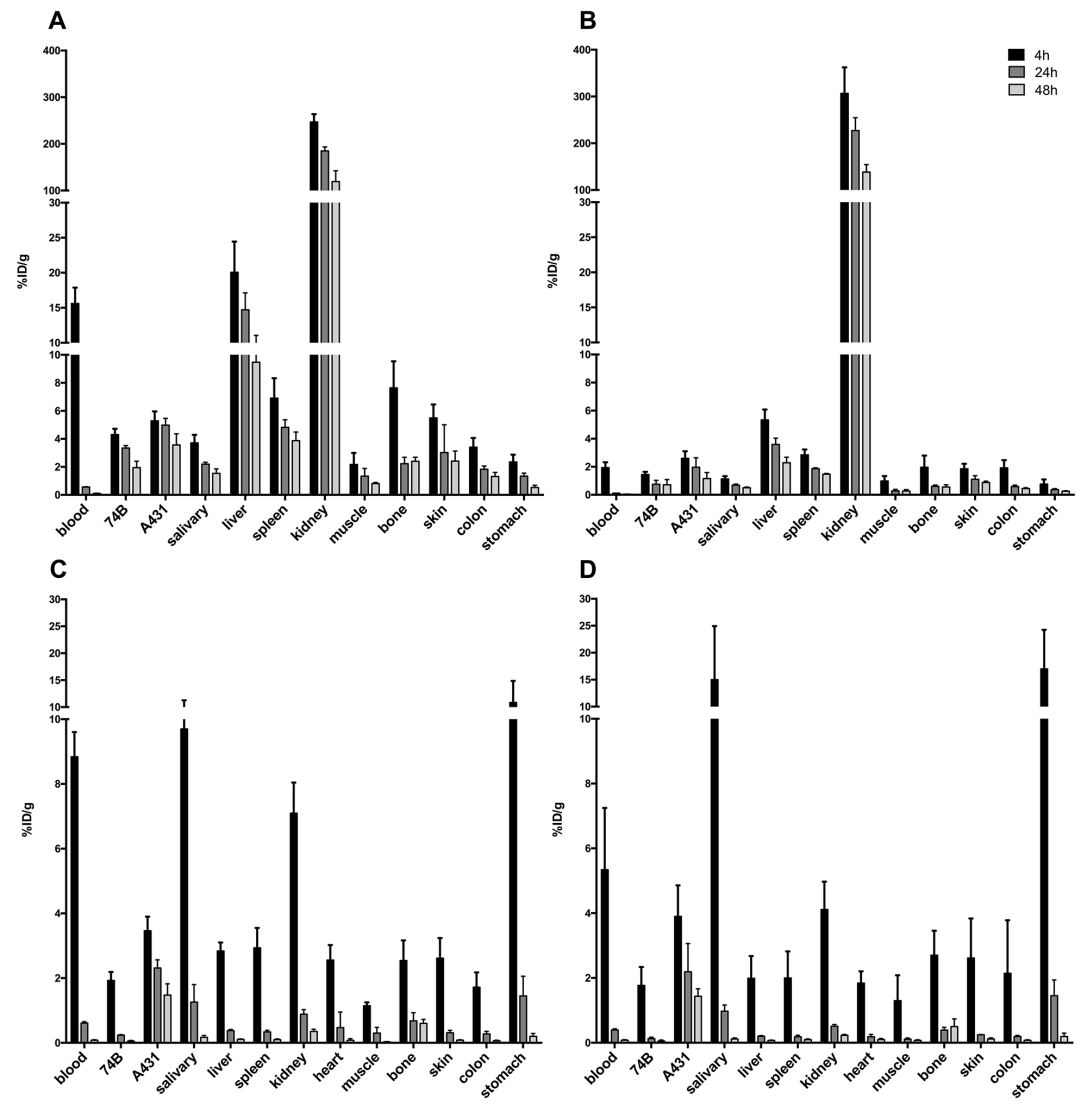
Uptake of radiolabeled scFv in tumor bearing mice, demonstrated as percent injected dose per gram (%ID/g) Error bars show standard deviation (N=4). **(A)** Uptake of ^111^In-DTPA-A11 in selected organs. **(B)** Uptake of ^111^In-DTPA-H12 in selected organs. **(C)** Uptake of ^125^I-A11(Iodogen) in selected organs. **(D)** Uptake of ^125^I-H12(Iodogen) in selected organs.

Distribution and uptake of^125^I-A11(Iodogen), ^111^In-DTPA-A11, ^125^I-H12(Iodogen), and ^111^In-DTPA-H12 was assessed *in vivo* by measuring uptake in mice bearing both CD44v6 low- (UM-SCC-74B) and CD44v6 high-expressing tumors (A431). Uptake calculated as%ID/g in selected organs is presented in Figure 4A-4D.

The uptake (%ID/g) of ^111^In-DTPA-A11 (Figure 4A) was highest at 4 h in all organs and decreased over time, with the most rapid decrease observed for blood, where activity decreased from 15.6 ± 2.3%ID/g at 4 h to 0.6 ± 0.02 ID/g 24 h p.i.. ^111^In-DTPA-H12 (Figure 4B) demonstrated a similar pattern with a maximum uptake at 4 h p.i. in tumors and other organs, and decrease in activity over time to 48 h. The organs with the highest uptake measured were kidneys, bone, skin and liver at 4 h p.i for both ^111^In-DTPA-A11 and ^111^In-DTPA-H12. For both conjugates, uptake was higher in the high CD44v6-expressing A431 tumors compared to low CD44v6-expressing UM-SCC-74B tumors. However, this difference was not statistically significant except for ^111^In-DTPA-A11 at 24 and 48 hours p.i.

Both ^125^I-A11(Iodogen) and ^125^I-H12(Iodogen) exhibited a more rapid organ clearance over time compared to their ^111^In-labeled counterparts, most markedly between 4 hours p.i and 24 hours p.i. (Figure 4). At 4 hours p.i. uptake was higher in blood, salivary gland, kidneys and stomach as compared to A431 tumors. However, already at 24 hours p.i., uptake in A431 tumors was higher than in any other organ (2.31 ± 0.21%ID/g and 2.18 ± 0.87%ID/g for ^125^I-A11(Iodogen) and ^125^I-H12(Iodogen), respectively, with the exception of thyroid (%ID/organ) in which activity uptake remained stable over time (data not shown). Both ^125^I-A11(Iodogen) and ^125^I-H12(Iodogen) exhibited a significantly lower uptake in low CD44v6-expressing UM-SCC-74B tumors compared to high CD44v6-expressing A431 tumors at all assessed time points.

**Figure 5:**
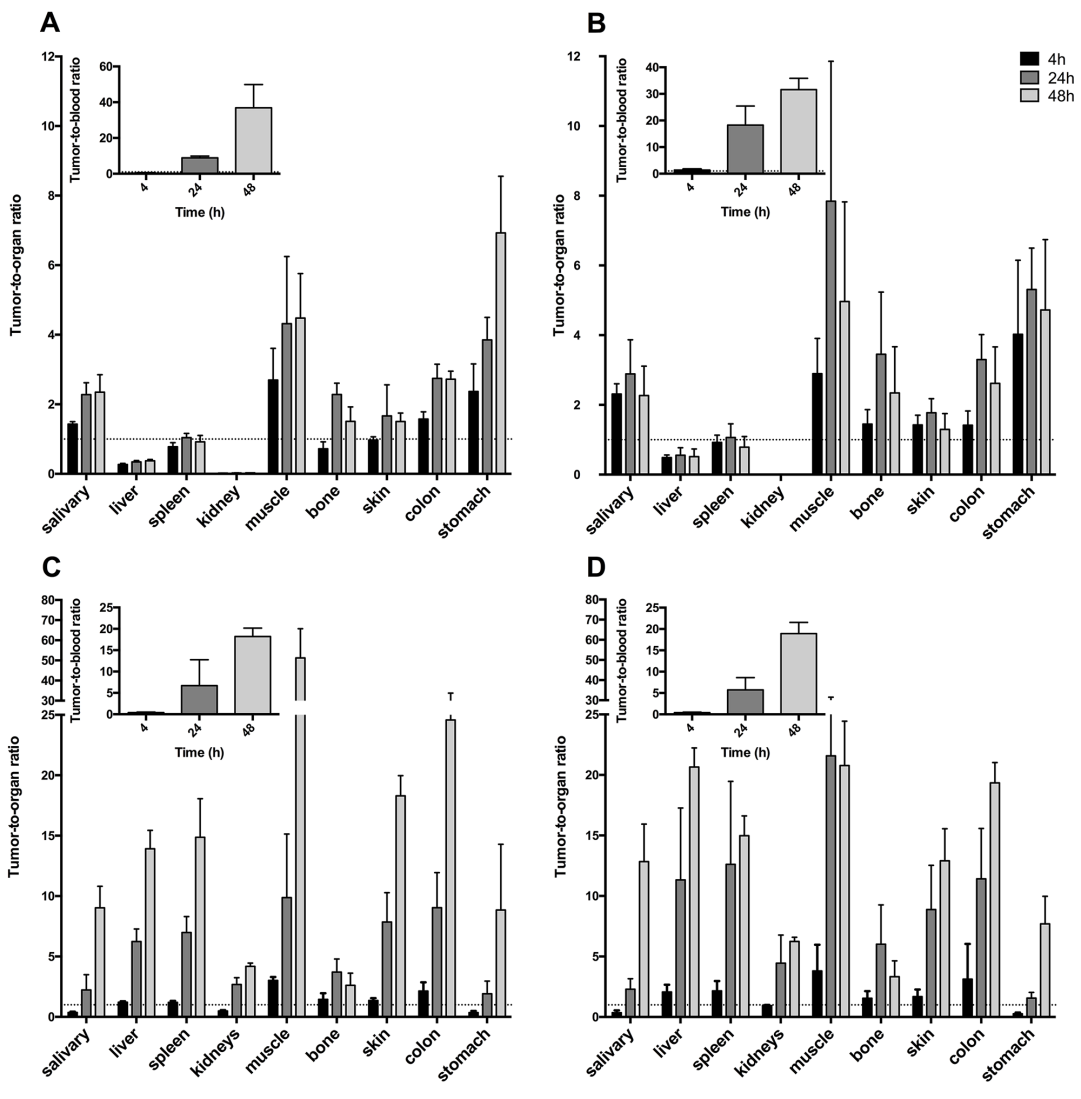
A431 tumor-to-organ ratios for selected organs Inset: Tumor-to-blood ratios. Error bars show standard deviation (N=4). **(A)** Tumor-to-organ ratios for ^111^In-DTPA-A11. **(B)** Tumor-to-organ ratios for ^111^In-DTPA-H12. **(C)** Tumor-to-organ ratios for ^125^I-A11(Iodogen). **(D)** Tumor-to-organ ratios for ^125^I-H12(Iodogen).

Tumor-to-organ ratios were calculated for the high CD44v6 expressing tumors (A431) (Figure 5A-5D) in order to evaluate the contrast of the radioactive signal between tumors and surrounding tissue and blood. Tumor-to-organ ratios of ^111^In-DTPA-A11 and ^111^In-DTPA-H12 (Figure 5A-5B) followed the same pattern as biodistribution data, with low ratios for kidneys, spleen, bone and liver. Tumor-to-organ ratios increased from 4 hours p.i. to 24 hours p.i. but remained stable or decreased to 48 hours p.i. Tumor-to-blood ratios increased with time and reached 8.8 ± 1.0%ID/g and 18.2 ± 7 for ^111^In-DTPA-A11 and ^111^In-DTPA-H12 respectively at 24 hours. At 48 hours p.i., tumor-to-blood ratios were 36.9 ± 13.0 for ^111^In-DTPA-A11 and 31.6 ± 4.3 for ^111^In-DTPA-H12.

Tumor-to-organ ratios of ^125^I-A11(Iodogen) and ^125^I-H12(Iodogen) can be seen in Figure 5C and 5D. While tumor to organ ratios were low already at 4 h p.i. (below 1 in most organs), both ^125^I labeled fragments displayed ratios well above 1 in all organs, including blood, from 24 h p.i. (Figure 5C and 5D). Tumor to blood ratio was 6.7±6.0 for ^125^I-A11(Iodogen) and 5.7±2.8 for ^125^I-H12(Iodogen) at 24 h. At 48 h p.i. (darker grey bars in Figure 5C and 5D), contrast to surrounding tissue were even higher for most organs, with e.g. tumor-to-blood ratios of 18.2 ± 2.0 for ^125^I-A11(Iodogen) and 18.9 ± 2.6 for ^125^I-H12(Iodogen), suggesting an increased sensitivity for tumor detection.

**Figure 6:**
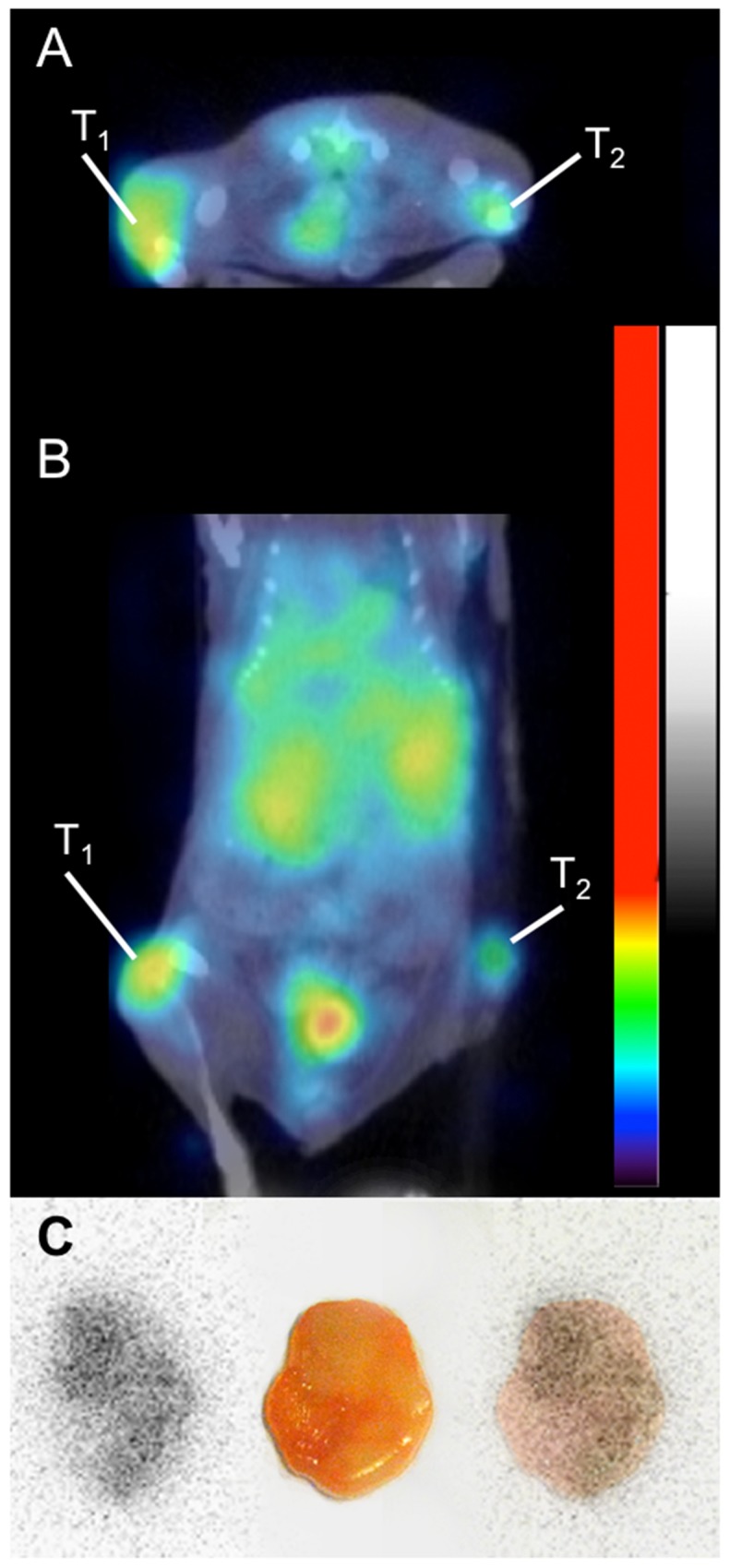
Small animal PET-CT imaging of 124I-H12(CAT) at 48 hours p.i., demonstrating a high CD44v6-expressing tumor (A431) on the left flank (T1) and a low CD44v6-expressing tumor (UM-SCC-74B) on the right flank (T2), transaxial view **(A)** and coronal view **(B)**. Besides tumor uptake, activity in urinary bladder and kidneys can be seen. **(C)** Digital autoradiography of the high CD44v6-expressing tumor (left), photo (middle) and superimposed image (right).

A small animal PET-CT study was also performed 48 h p.i. using ^124^I-H12(CAT) for proof-of-concept (Figure 6A and 6B). The high CD44v6-expressing A431 tumor was clearly visible, whereas the low CD44v6-expressing UM-SCC-74B tumor was faintly visible. Kidneys and bladder were also visible in the image. Digital autoradiography of the high CD44v6-expressing A431 tumor demonstrated homogenous intratumoral distribution of the radioactivity (Figure 6C).

## DISCUSSION

[^18^F]FDG-PET is widely used for staging unknown primary tumor localization, and post treatment follow up in patients affected by HNSCC. The major limitation of [^18^F]FDG is that the metabolic uptake mechanism is not specific to the disease, resulting in undesired additional uptake in inflammatory and treated tissue. Thus there is an unmet need for the development of an imaging agent that would demonstrate specific binding to HNSCC and allow accurate quantification for treatment response evaluation. Antibody-based molecular imaging would add specificity to the evaluation of post treatment HNSCC, and a favorable target for this application may be CD44v6. Previous studies have demonstrated promising targeting potential in HNSCC patients using a ^99m^Tc-labeled full-length CD44v6-targeting antibody [30]. Full-length antibodies are however large, typically show slow clearance from blood and non-target tissues and do not penetrate tumors as efficiently as smaller fragments. This can delay the time point for imaging, and often result in sub-optimal contrast between tumor and surrounding normal tissue. Therefore, smaller molecules with retained affinity and improved pharmacokinetics are believed to improve contrast between tumor and tissue within a timeframe better suited for clinical use. Furthermore, by using *in vitro* antibody libraries instead of traditional immunization combined with the hybridoma technology, it is possible to monitor binding specificity and characteristics throughout the selection process to promote generation of binders with sought properties. In addition, the recombinant nature of *in vitro* antibody libraries is well adopted for subsequent modifications of the isolated genes with traditional cloning methods and further optimizations.

Consequently, the objective of the present study was to select novel CD44v6-targeting scFv antibodies to CD44v6 and to evaluate their potential as imaging probes in HNSCC. Two high affinity variants, CD44v6-scFv-A11 and CD44v6-scFv-H12 were generated by chain-shuffling of a subset of clones initially selected from a naïve scFv library by phage display. Both variants displayed sub-nanomolar affinities to human CD44v6 with no observed cross-reactivity to a closely related CD44 isoform that lacks the region of interest. By using peptides spanning different regions of the v6 sequence, the epitope could be mapped to a 14-residue stretch. No binding was observed to peptides that included the seven N- or C-terminal residues of this region, respectively. Interestingly, the here presented selections on an antigen that contained the full v6 region enriched binders to the same shorter region that we have previously used to select CD44v6-binding antibodies that display promising properties in imaging applications [27, 28]. Moreover, the same motif has been implicated in co-receptor function of CD44v6 for c-Met and VEGFR-2 [31]. Similarly to the previously described Fab fragment AbD15179 no cross-reactivity was observed to a rat CD44v6 peptide, which can probably be attributed to the relatively low sequence homology (43%) in this region.

The two selected top candidates CD44v6-scfv-A11 and CD44v6-scfv-H12 were subsequently labeled with ^111^In and ^125^I in order to create radio-immunoconjugates suitable for molecular imaging using PET or SPECT, with ^125^I as a model for ^124^I. Two radiolabeling methods were assessed for each radionuclide, rendering eight imaging agent candidates that were subjected to biological evaluation in terms of stability, binding specificity and kinetics *in vitro*. For ^111^In labeling, two chelator moieties (DOTA and DTPA) were assessed, yielding ^111^In-DOTA-A11, ^111^In-DOTA-H12, ^111^In-DTPA-A11 and ^111^In-DTPA-H12. Stability assays using challenge with EDTA revealed somewhat more stable conjugates for ^111^In-DOTA-A11 and ^111^In-DTPA-A11 than for ^111^In-DOTA-H12 and ^111^In-DTPA-H12. Furthermore ^111^In-DTPA-H12 demonstrated higher stability than ^111^In-DOTA-H12. For ^125^I labeling, two direct labeling methods, CAT and Iodogen, were performed, yielding ^125^I-A11(CAT), ^125^I-H12(CAT), ^125^I-A11(Iodogen), and ^125^I-H12(Iodogen). Both iodination methods provide regiospecific labeling at tyrosine amino acid residue, although the Iodogen method allows milder conditions, which can be crucial for sensitive macromolecules. On the other hand, CAT labeling often provides higher specific activity, which is an advantage in the imaging setting. Even though the CAT method presents harsher oxidizing conditions than Iodogen, the cell binding capability of CD44v6-scFv-H12 was preserved using both ^125^I and ^124^I. Thus for CD44v6-scFv-H12, CAT labeling may be preferable for imaging applications. However, CD44v6-scFv-A11 proved to be more sensitive to iodination conditions, as a clear difference in cellular binding could be observed between the two methods in real time assays using LigandTracer. ^125^I-A11(Iodogen) displayed a clear binding signal whereas ^125^I-A11(CAT) did not (Figure 2A), even though specific activity values of the tracers were similar. It should be noted that CD44v6-scFv-A11 has one unique tyrosine residue in CDRL1, which could have an effect on the binding upon labelling. This demonstrates the importance of labeling optimization and the potential impact of labeling method on the biological function of the resulting tracer.

After the initial selection based on the tracer production efficiency, stability and target binding capability of the resulting tracer candidates, the four candidates ^111^In-DTPA-A11, ^111^In-DTPA-H12, ^125^I-A11(Iodogen), and ^125^I-H12(Iodogen) were further evaluated for head-to-head comparisons *in vitro* and *in vivo*. The binding kinetics to cellular CD44v6 studied in real time using LigandTracer revealed similar binding patterns for the four tracers, with fast on-rates, followed by an initially fast dissociation during subsequent 20 min, succeeded by a slower dissociation for the remainder of the assay with off-rates of 1.44*10^-5^ s^-1^ and lower (Figure 2B). Signal intensities varied between conjugates even though specific activities were similar, with higher target signals on A431 cells obtained for the CD44v6-scFv-H12 conjugates compared to their CD44v6-scFv-A11 counterparts (Figure 2C). As expected, the uptake was higher on the high CD44v6-expressing A431 cells, whereas signals barely above the detection limit of the instrument were obtained on low CD44v6-expressing UM-SCC-74B cells, indicating that CD44v6-specific binding correlate to target abundance in the investigated sample (Figure 2C). Blocking assays verified LigandTracer results, with an excess of unlabeled fragment significantly reducing the binding of the labeled fragments, albeit to a higher extent for the CD44v6-scFv-H12 conjugates compared to the CD44v6-scFv-A11 counterparts (Figure 2D). It should be noted that these blocking assays are endpoint assays, not taking into account if binding equilibrium has been reached, or potential loss of binders in the subsequent washing steps. This might account for some of the lower cell binding on unblocked cells, and consequently lower blocking ability obtained for ^125^I-A11(Iodogen) and ^111^In-DTPA-A11.

Next, an organ distribution study in tumor bearing mice was designed for the four tracers ^111^In-DTPA-A11, ^111^In-DTPA-H12, ^125^I-A11(Iodogen), and ^125^I-H12(Iodogen). A dual-nuclide, dual-tumor setup was used in order to reveal differences dependent on either the scFv fragments (CD44v6-scFv-A11 versus CD4v6-scFv-H12) or radiolabeling modifications (chelator-mediated labeling with ^111^In versus direct labeling at tyrosine residues with ^125^I). In this experimental model, ^111^In-DTPA-A11 together with ^125^I-A11(Iodogen), or alternatively ^111^In-DTPA-H12 together with ^125^I-H12(Iodogen), in a total amount of 3 μg tracer per animal, was evaluated simultaneously in the same mouse, each mouse bearing both a low CD44v6-expressing UM-SCC-74B tumor and high CD44v6-expressing A431 tumor. By using this model, evaluations of biodistribution, tumor uptake, contrast, as well as specificity and sensitivity of the tracers could be performed while eliminating inter-subject variability and potential bias in e.g. tumor size or injection variability. The quality of the xenografted tumors and potential *in vivo* effect on CD44v6-expression was first assessed *ex vivo* with IHC analysis of A431- and UM-SCC-74B xenografted tumors (Figure 3). IHC stainings confirmed the high CD44v6 expression on A431 tumors, and the lower, albeit clear, cell surface expression of CD44v6 on UM-SCC-74B tumors. This verified the use of the dual-tumor setup for tracer specificity evaluation *in vivo*.

Clear tracer specificity was demonstrated *in vivo* for both iodinated tracers (^125^I-A11(Iodogen) and ^125^I-H12(Iodogen)), exhibiting significantly lower uptake in low CD44v6-expressing UM-SCC-74B tumors compared to high CD44v6-expressing A431 tumors at all assessed time points. Also for ^111^In-DTPA-A11 and ^111^In-DTPA-H12 uptake was higher in A431 tumors compared to UM-SCC-74B tumors, although this difference was not statistically significant at all time points. Thus, the iodinated tracers were better able to discriminate between high- and low- CD44v6-expressing tumors in these settings, and both CD44v6-scFv-A11 and CD44v6-scFv-H12 demonstrated *in vivo* specificity (Figure 4). As for the radioactivity fraction associated with blood (%ID/g), a faster blood clearance was observed for the^125^I conjugates compared to the ^111^In-labelled conjugates. This is in line with previous studies by our group, where CD44v6-targeting Fab and Fab_2_ fragments were evaluated [27, 28]. For ^125^I-A11(Iodogen) and ^125^I-H12(Iodogen), activities in blood were in the same range. However, for ^111^In-labeled conjugates, this value was significantly higher for ^111^In-DTPA-A11 compared to ^111^In-DTPA–H12 (Figure 4). This could indicate that the ^111^In-DTPA-A11 tracer was more stable *in vivo* than ^111^In-DTPA-H12, which is in line with the *in vitro* stability assays on ^111^In-labeled conjugates.

Biodistribution and tumor-to-organ ratios (Figures 4 and 5) demonstrated a more favorable distribution for the iodinated compounds compared to the indium labeled ones. Already from 24 h p.i., the ^125^I labeled fragments displayed tumor-to-organ ratios above one in all organs, whereas the ^111^In-labelled conjugates demonstrated less desirable tumor-to-organ ratios due to the higher uptake in liver, spleen and kidneys. The CD44v6 receptor does not actively internalize, which could favor a radiohalogen compared to a radiometal, where a radiometal may be more suitable for internalizing receptors due to its residualizing properties [32]. High uptake of both ^111^In-DTPA-A11 and ^111^In-DTPA-H12 in liver is in accordance with previous reports and could be due to liver digestion or interaction with metal binding proteins such as iron-binding proteins [33]. The size of scFvs is below the renal threshold, and thus the elimination via the kidneys is the main pathway for clearance. Correspondingly, high uptake in kidneys was seen for all four conjugates initially at 4 h p.i.. The retained activity of ^111^In conjugates at later time points could be caused by binding of ^111^In to proteins in the tubuli [33, 34], which might limit clinical use of the ^111^In-labeled conjugates. However, since HNSCC rarely metastasizes to kidneys and more commonly to liver, renal clearance would be preferable as high uptake in liver could disguise disease. As expected, activities in thyroid, measured as%ID/organ, were higher for the iodinated tracers and are most likely due to uptake of catabolites and free iodine as demonstrated in previous studies [29]. Initial high uptake in stomach and salivary glands would also suggest ^125^I catabolites and transport of those into tissues where Na^+^ /I^–^pumps are readily available [35]. Uptake of iodine catabolites can however be blocked by administration of NaI or KI pre-injection if necessary [28]. The proof-of-concept small animal PET-CT study was in line with the biodistribution data, clearly visualizing the high CD44v6-expressing A431 tumor, and digital autoradiography demonstrated homogenous distribution of the radioactivity within the A431 tumor (Figure 6A-6C). By further optimizing e.g. labeling methods, specific activities, doses and imaging time points, tumor visualization and contrast to non-tumor tissue could be further improved. Furthermore, it would be of interest to also assess other radionuclides with potential for imaging, such as ^89^Zr and ^67^Ga.

Tumor uptake peaked at 4 h p.i. for all tracers, and tumor-to-blood ratios were similar, exceeding 5 at 24 h p.i. and 25 at 48 h p.i.. This tumor-to-blood contrast is superior to what has been obtained in previous studies, where a CD44v6-targeting Fab fragment, AbD15179 labeled with ^125^I, reached a tumor-to-blood ratio of 1.5 at 24 h and around 4 at 48 h p.i [28], and a following study using the bivalent AbD19384 labeled with ^125^I showed a tumor-to-blood ratio maximum of above 4 at 72 h p.i. [27]. Thus, already at 24 h p.i. our scFv fragments provided superior tumor-to-blood ratios than obtained with the Fab and Fab_2_ fragments at 48 and 72 h p.i.. Compared to other studies with radiolabeled molecules the tumor-to-blood ratios accomplished are competitive. Studies using monovalent scFv, bivalent scFv and scFv-Fc, where the Fc region is coupled to the fragment, report tumor-to-blood ratios varying between 3-14 at 24 hours p.i. [36, 37]. Tumor-to-blood ratios in studies using bivalent antibody fragments range 1 - 5 in the selected studies [38, 39].

The advantageous tumor-to-blood ratio of the radiolabeled scFv fragments that was achieved in this study could be due to the lower molecular weight, leading to faster clearance from blood. This combined with the sub-nanomolar affinities and slow target off-rates of the fragments would lead to very favorable tumor-to-blood ratios. This suggests that the present scFv fragments, if further optimized for suitable radionuclides, labeling techniques and dosing, could provide even higher imaging contrast, and at earlier time points, than previously assessed conjugates. However, for therapeutic purposes scFv are not optimal due to the fast clearance, which does not allow for long enough exposure to the tumor. This can be compared to full size antibodies, which are accumulated in tumors for 1-5 days [7, 8]. One way to increase the size, thereby limiting kidney elimination and increasing avidity, could be reformatting to other antibody formats, such as diabodies [12]. Furthermore, expression with a suitable fusion tag to prolong circulation times, etc. is also possible and can further expand the potential applications for these tracers [40]. If needed, resultant antibody clones may also be further improved with aspect to parameters as specificity, affinity and stability.

In overview, we have generated two novel high affinity variants, CD44v6-scFv-A11 and CD44v6-scFv-H12 by chain-shuffling of a subset of clones selected from a naïve scFv library by phage display. These fragments demonstrated subnanomolar dissociation equilibrium constants (K_D_), and specific human CD44v6-binding, with the epitope located within residues KEQWFGNRWHEGYR of CD44v6. Next, CD44v6-scFv-A11 and CD44v6-scFv-H12 were radiolabeled for potential imaging applications, using ^111^In and ^125^I. Several labeling methods were assessed, creating eight different conjugates that were evaluated *in vitro* on cultured CD44v6-expressing tumor cells. Seven of the labeled conjugates demonstrated CD44v6-specific binding, and the conjugates ^111^In-DTPA-A11, ^111^In-DTPA-H12, ^125^I-A11(Iodogen), and ^125^I-H12(Iodogen) were chosen for subsequent *in vivo* biodistribution studies. Tumor-to-blood ratios for all assessed conjugates were superior to those previously obtained with CD44v6-targeting Fab and Fab_2_ fragments. Iodinated scFv tracers were better able to discriminate between high- and low- CD44v6-expressing tumors than the indium-labeled counterparts, and displayed more favorable tumor-to-organ rations, exceeding one for all organs already at 24 h p.i.. Indium-labeled tracers demonstrated higher uptake in liver, spleen and kidneys, and biodistribution of ^111^In-DTPA-H12 suggested reduced *in vivo* stability for this conjugate. Thus, the iodinated conjugates were the most promising candidates for CD44v6-targeted molecular imaging in this study, and this was further verified in a proof-of-concept small animal PET-CT using ^124^I-labeled CD44v6-scFv-H12. To summarize, in this study novel CD44v6-binders were selected from a scFv phage display library and affinity maturated. The top candidates were radiolabeled and preclinically evaluated both *in vitro* and *in vivo* with iodinated conjugates demonstrating potential for imaging and quantification of target expression. The further investigation of the feasibility of monitoring response to therapy and evaluation of residual or recurrent disease in HNSCC is warranted.

## METHODS

### Protein antigens

If not stated otherwise, proteins used for antibody selection and characterization have been described previously [41].

### Phage display scFv selection

Antibodies were selected from a scFv phage display library by two rounds of selection using previously described procedures [42, 43]. Briefly, the antigen CD44v3-10 (5 μg/ml in PBS) was coated to Maxisorp tubes (Nunc Inc.) and a negative selection step was performed against Fc-fused CD44v3-10Δv6 prior to the first round.

### ELISA

For polyclonal scFv-phage ELISA, separate wells of 96-well Maxisorp plates (Nunc Inc.) were coated over night at 4°C with 2.5 μg/ml CD44v3-10 or CD44v3-10Δv6 along with three unrelated, negative control proteins, NRR1-Fc, NRR1-CD4, and Fc-His-FLAG (previously described [42]). Coated plates were washed with PBS and blocked with PBS supplemented with 2% non-fat dry milk (PBSM) before addition of rescued scFv-phage populations (diluted 1:4 or 1:20 relative to the initial culture volume in PBSM) for 1 hour at room temperature. Unbound phage were washed away with PBS supplemented with 0.05% Tween (PBST) and PBS prior to incubation with the phage specific antibody, mouse anti-M13 (1:500 in PBSM, GE Healthcare, # 61097), for 1 h at room temperature. Plates were washed as above and HRP conjugated anti-mouse antibody (1:1000 in PBSM, Thermo Scientific) was added for 1 h. Bound scFv-phage were detected by the HRP substrate TMB (Sigma-Aldrich, #T4444) and absorbance measured at 450 nm in a plate reader according to manufacturer’s recommendations.

To screen for individual scFv antibodies with ELISA, selected phage populations were subcloned and individual scFv clones expressed in 96-well plates as previously described [42]. Culture supernatants from individual scFv clones were used for ELISAs as described previously [42] with the exceptions that clear Maxisorp plates were coated with 50 μl (2.5 μg/ml) CD44v3-10 or CD44v3-10Δv6 followed by an anti-FLAG-HRP antibody (Sigma-Aldrich, #A8592) and TMB substrate to detect bound scFv protein by measuring the absorbance at 450 nm.

### Affinity maturation

For affinity maturation, V_H_ chains from 25 unique scFv clones were rearranged with a naive V_L_ library through a chain shuffling strategy to generate a diversified library. Selected V_H_ sequences were isolated by PCR using the primers (CGCTGCCCAGCCGGCCATGG) and (CTAGCGCCACCGCCAGA). Amplified V_H_ fragments were pooled and cloned en masse into the *NcoI* and *XhoI* sites of a phagemid vector harboring a naïve V_L_ repertoire [43]. The resulting CD44v6-scFv library was transformed into electrocompetent TG1 *E. coli* cells. Insertion of V_H_ fragment was confirmed by colony-PCR.

The shuffled CD44v6-scFv phage library was rescued as previously described [43] and used for selection of affinity matured scFv clones. In total four rounds were performed. A first round to enrich for clones binding CD44v6 was done by solid phase biopanning against CD44v3-10 coated to a Maxisorp tube. Subsequent rounds were performed in solution with biotinylated CD44v3-10 and capture of bound phage on magnetic beads (Biotin binder, Invitrogen). Soluble selections were carried out for three rounds (round 2 - 4) using CD44v3-10 concentrations ranging from 100 pM – 0.1 pM. Rounds 3 and 4 included extended washing times (30 minutes at room temperature) before elution. Selected phage populations were subcloned into pSANG10-3F [43] and transformed to BL21(DE3) *E. coli* cells. Individual colonies were transferred to four 96-well culture plates and stored as glycerol stocks.

### scFv production

For kinetic studies, cell binding and *in vivo* experiments, scFv clones were produced by inoculating shake flasks containing 50 - 100 ml 2YT media (supplemented with 50 μg/ml kanamycin) with the *E. coli* strain BL21(DE3) harboring the individual scFv clones in the expression vector pSANG10-3F. When cultures reached OD600 = 0.8 - 1, expression was induced by addition of 1 mM isopropyl-beta-D-1-thiogalactopyranoside (IPTG) and, following over night incubation at +30°C, scFv were recovered from the periplasm as previously described [44]. Periplasmic fractions were further affinity purified (Nickel affinity gel, Sigma-Aldrich, #P6611). Unbound material was washed away with washing buffer (PBS supplemented with 20 mM imidazole and 150 mM NaCl) before the purified antibodies were eluted in elution buffer (PBS supplemented with 400 mM imidazole and 150 mM NaCl). Protein-containing fractions were pooled and dialyzed against PBS (dialysis kit, Sigma-Aldrich, #PURD35050) and analyzed by SDS-PAGE. Concentrations of recovered antibodies were determined by A280.

### Surface plasmon resonance (SPR)

Recombinant CD44v3-10 and the v6-negative isoform of CD44 (CD44v3-10Δv6) [41], were imm-obilized by standard amine coupling on a ProteOn XPR36 general layer medium sensor chip (Bio-Rad Laboratories, Hercules, CA, USA) to immobilization levels of 750-2700 (four separate surfaces) and 2500 RU, respectively. Dilution series ranging from 1.6-100 nM of scFv in PBST (pH 7.4) were injected at 50 μl/min at 25°C. Signal from a blank surface and a parallel buffer injection were subtracted and binding data fitted to a 1:1 Langmuir model using ProteOn Manager software version 3.1.0.6 (Bio-Rad Laboratories). Kinetic constants were calculated as average values from several (2-8) measurements using two dilution series (2-fold dilutions starting from 50 and 100 nM, respectively) and up to four different ligand densities. Surfaces were regenerated with 10 mM HCl between injections. To enable mapping of binding to a shorter region in the v6 region, three overlapping peptides spanning 20 amino acid regions (residues 1-21, 12-32 or 22-43 of v6; United Biosystems) were conjugated to bovine serum albumin (BSA) and immobilized by amine coupling to ca 4000 RU to a separate chip as described above. Moreover, to test species specificity and narrow down the epitope region, competition experiments with human or rat 14-mer peptides [27]) were carried out. Briefly, signal from injection of scFv alone (50 nM) on a surface with immobilized CD44v3-10 was compared to scFv pre-incubated with a 10-fold molar excess or peptide prior to injection.

### Cell lines

The human squamous cell carcinoma (SCC) cell line A431 (obtained from American Type Culture Collection) was cultured in Ham’s F10, supplemented with 10% fetal calf serum, 2 mM L-glutamine, and antibiotics (100 IU penicillin and 100 µg/ml streptomycin). A431 has been shown to be a highly CD44v6-expressing cell line with approximately 3x10^6^ antigens per cell [45]. The human SCC cell line UM-SCC-74B (kindly provided by Professor TE Carey, University of Michigan, USA) was cultured in DMEM with the same supplements as well as 1% non-essential amino acids. UM-SCC-74B has demonstrated low expression of CD44v6 (approximately 0.1x10^5^ antigens per cell). Cells were incubated at 37°C in an atmosphere containing 5% CO_2_.

### Labeling of scFv

#### Iodination using chloramine T

scFvs CD44v6-scFv-A11 and CD44v6-scFv-H12 were first labeled with ^125^I (PerkinElmer, Waltham, MA, USA using direct chloramine T labeling (CAT) [46] (Sigma-Aldrich). CAT and Na_2_SO_5_ (NBS) were dissolved in MilliQ-water to 4 mg/ml. Fifteen µg of CD44v6-scFv-A11 or CD44v6-scFv-H12 in PBS was added to 5 MBq of ^125^I and mixed before adding 10 µl of CAT (4 mg/ml in PBS), and the volume was adjusted with PBS to 130 µl. The reaction mixture was incubated for 1 min on ice before ending the reaction by adding 20 µl of NBS (4 mg/ml in PBS). For small animal PET-CT imaging of ^124^I-labeled CD44v6-scFv-H12, approximately 32 MBq ^124^I was incubated for 20 minutes with NaI (in a 2:1 ratio with CD44v6-scFv-H12) before the addition of 40 µg of CD44v6-scFv-H12 (0.151 mg/ml) and 20 µl of CAT (4 mg/ml). The reaction was incubated on ice for 60 s before ending the labeling process by the addition of 40 µl of NBS (4 mg/ml). Samples were purified on a NAP5 size exclusion column (GE Healthcare, Uppsala, Sweden) equilibrated with PBS. Conjugates iodinated using CAT are referred to as “^125^I-A11(CAT)”, “^125^I-H12(CAT)”, and “^124^I-H12(CAT)” in this paper.

#### Iodination using Iodogen

Labeling using Iodogen, 1,3,4,6-Tetrachloro-3α,6α-diphenylglycouril, was performed as follows; three Iodogen buffers (A, B, C) were prepared; A) 0.5 M sodium phosphate buffer, pH 7.4., B) 0.05 M sodium phosphate and 5 M NaCl, pH 7.4., C) 0.05 M sodium phosphate, 5% potassium iodide and 0.5% BSA (w/v). All buffers were prepared using MilliQ-water. Iodogen was dissolved in dichloromethane to 0.1 mg/ml*.* ^125^I and 40 µg of CD44v6-scFv-A11 or CD44v6-scFv-H12 in PBS (0.203 mg/ml and 0.151 mg/ml respectively) were mixed in a tube previously coated with 20 µg Iodogen. Buffer A was added in an equivalent volume, incubated at room temperature for 7 minutes and agitated carefully every 30 s using a vortex. The reaction mixture was transferred to a new tube and buffer B (1 ml) was subsequently added. Following at least 10 minutes of rest, buffer C (1 ml) was added, and the sample was mixed thoroughly. Labeled conjugates were separated from non-reacted radionuclide and low molecular weight reaction components by using a NAP-5 or PD10 column pre-equilibrated with PBS. Conjugates labeled with ^125^I using Iodogen are referred to as “^125^I-A11(Iodogen)” and “^125^I-H12(Iodogen)” in this paper.

#### ^111^In labeling using DOTA

Borate buffer (0.5 ml, 0.08 M, pH 9) was added to *N*-hydroxysulfosuccinimide ester of DOTA under stirring. The pH was further adjusted to 9.0 by adding borate buffer. The mixture was then immediately added to the antibody fragment solution (CD44v6-scFv-A11: 2.9 nanomoles; CD44v6-scFv-H12: 1.1 nanomole), stirred and left at room temperature for 3–4 h. The activated DOTA ester was added in 20-fold excess over the antibody fragments. Purification of the resulting bioconjugates was performed using centrifugal filter units (Ultracel YM-10; cutoff: 10000 Da). The recovery of the bioconjugate from the centrifugal filter unit was over 90% as determined by UV-HPLC. The separation of the analytes was accomplished on a size exclusion gel filtration column (Superdex peptide, 10/300, GE Healthcare) with mobile phase of 100 mM KCl and 25 mM K_2_HPO_4_ mixture (pH 8.0) under isocratic condition with flow rate of 1.0 ml/min. Data acquisition and handling were performed using the EZChrom Elite Software Package. The concentration of the antibody fragments and their bioconjugates was determined using DeNovix DS-11 Spectrophotometer (DeNovix, Wilmington, USA). Thereafter, 100 MBq of ^111^In in acetate buffer was added to 76 µg of DOTA-CD44v6-scFv-A11 bioconjugate (96 µl, 0.79 mg/ml), and 50 MBq ^111^In was added to 27 µg of DOTA-CD44v6-scFv-H12 bioconjugate (97 µl, 0.28 mg/ml), and the pH was adjusted to approximately 4.6 by adding acetate buffer (0.2 M, pH 5.5). The reaction mixtures were incubated for 10 min at 40°C. Labeled fragments were separated from non-reacted radionuclide and low molecular weight reaction components using a NAP-5 column pre-equilibrated with PBS. Conjugates labeled with ^111^In using DOTA ester are referred to as “^111^In-DOTA-A11” and “^111^In-DOTA-H12” in this paper.

#### ^111^In labeling using CHX-A”-DTPA

Labeling of CD44v6-scFv-A11 and CD44v6-scFv-H12 with ^111^In (Mallinckrodt Medical B.V.) using CHX-A”-DTPA was performed as described previously [47, 48]. In short, to 281 µg CD44v6-scFv-A11 or 184 µg CD44v6-scFv-H12 fragment solution (2.4 and 1.6 mg/ml respectively in borate buffer, pH 9), 31 µl or 20 µl of CHX-A”-DTPA solution (1 mg/ml in borate buffer, 0.07 M, pH 9) was added, corresponding to a chelator-to-scFv molar ratio of 5:1. The reaction mixture was incubated overnight at room temperature, and the conjugated scFv was separated from free CHX-A”-DTPA using a NAP-5 column equilibrated with acetate buffer (0.2 M, pH 5.5). Protein concentration on separated fractions was measured using a DeNovix DS-11 Spectrophotometer. Approximately 40 MBq of ^111^In in acetate buffer was added to 98.6 µg DTPA-CD44v6-scFv-A11 (170 µl, 0.58 mg/ml), and 25 MBq ^111^In was added to 56.8 µg DTPA-CD44v6-scFv-H12 (160 µl, 0.36 mg/ml), and the pH was adjusted to 4.5 by adding 20 µl acetate buffer (0.2 M, pH 5.5). The reaction mixtures were allowed to incubate during 30 min at room temperature. Conjugates labeled with ^111^In using CHX-A”-DTPA are referred to as “^111^In-DTPA-A11” and “^111^In-DTPA-H12” in this paper.

### Quality assessment of labeling

To determine the purity and stability of the labeled antibody fragments over time, instant thin-layer chromatography (ITLC) was performed on radiolabeled conjugates. Samples taken immediately after the labeling procedure, as well as stored in PBS at 4°C for 24 hours, were analyzed. Furthermore, stability of ^111^In-labeled conjugates was also evaluated with ITLC by challenge with 500-fold excess of EDTA at 37°C for 0.5 and 24 h. Approximately 0.5 µl of the conjugate was placed on a chromatography strip (Biodex) and put into a running buffer (70% acetone or 0.2 M citric acid for ^125^I/^124^I and ^111^In, respectively), followed by measurements on a Cyclone Storage Phosphor System. Data were analyzed using the OptiQuant image analysis software.

### Cell studies *in vitro*

#### Flow cytometric analysis

A431 cells were dissociated with trypsin, washed in DMEM containing 10% FBS and transferred to fresh tubes (10^5^ cells/ tube). Cells were pelleted by gentle centrifugation and re-suspended in 50 µl PBS containing 0.5% BSA (PBSB). Next, 5-50 µl of respective scFv clone were added to separate tubes (scFv concentrations varied between 1-20 µg/ml in this experiment) and final volumes adjusted to 100 µl before incubation 1 h. Cells were washed with 3x1ml PBSB and incubated with 30 µl (10 µg/ml) mouse-anti-FLAG antibody (Sigma Aldrich, #F9291) for 30 minutes. Following additional wash, bound antibody was probed with 30 µl (20 µg/ml) Alexa488-conjugated goat anti-mouse antibody (Thermo Scientific, #A-11001) for 30 minutes. Flow cytometer analysis (Beckman Coulter, Gallios) was then used to then rank CD44v6-scFv clones according to the capability to bind CD44v6 on A431 cells.

### *In vitro* binding measurements on cultured tumor cells

Real-time *in vitro* binding and retention measurements of the radiolabeled conjugates were performed at room temperature using LigandTracer instruments (Ridgeview Instruments AB, Uppsala, Sweden) on A431 (high CD44v6-expression) cells and UM-SCC-74B (low CD44v6-expression) cells, approximately 5x10^6^ cells at the time of experiment. LigandTracer Grey was used for ^125^I-labeled conjugates and LigandTracer Yellow was used for ^124^I- and ^111^In-labeled conjugates. Generally, binding traces using several subsequent concentrations (0 – 9 nM) were obtained for at least 1 h per concentration, followed by a dissociation measurement for at least 10 h. The shapes of the real-time binding curves produced in LigandTracer were compared in the evaluation software TraceDrawer 1.6.1 (Ridgeview Instruments AB, Vänge, Sweden).

### Blocking assay

Blocking assays were performed on ^125^I-A11(Iodogen), ^125^I-H12(Iodogen), ^111^In-DTPA-A11 and ^111^In-DTPA-H12. Dishes seeded with A431 cells two days before experiments were washed with serum free medium. Radiolabeled conjugate was added at a concentration of 10 nM. In order to determine binding specificity, 100-times molar excess of non-labeled binder was added to block binding of labeled conjugate. The samples were allowed to incubate for 4 hours at 37°C, 5% CO_2_. The incubation medium was removed, and the cells were washed with serum free medium. The cells were detached with trypsin-EDTA at 37°C, and resuspended in complete medium. A sample was taken for cell counting and the remaining solution was collected for radioactivity measurements in a gamma well counter (1480 WIZARD; Wallace Oy).

### Small animal studies

Female nu/nu Balb/c mice were housed under standard laboratory conditions and fed *ad libitum*. All experiments complied with Swedish law and were performed with permission from the Uppsala Committee of Animal Research Ethics.

#### Immunohistochemistry

Immunohistochemistry (IHC) was performed on six A431 and UM-SCC-74B tumors, fixated in formalin directly after dissection. Tumors were paraffin-embedded, sectioned and deparaffinized. Antigen retrieval was achieved by microwaving (10+15 min) in citrate buffer (DAKO, S2369) or Tris-EDTA buffer (DAKO, S2367). Immunostaining with anti-CD44v6 (AbD Serotec) was performed according to manufactures instructions followed by detection with the EnVision FLEX system (DAKO, K8000). The reaction was visualized by EnVision FLEX DAB+ (DAKO). Mayer’s hematoxylin (DAKO) was used as counterstain. Images of the IHC staining (magnification x10) were obtained using a Nikon D3000 digital camera mounted on an inverted Nikon Diaphot-TMD 290 microscope.

#### Biodistribution studies

In total 25 mice were used to analyze *in vivo* binding of radiolabeled fragments to xenografts with varying CD44v6 receptor densities. Approximately 8x10^6^ A431 cells (high CD44v6 expression) suspended in 200 µl cell medium were injected subcutaneously into the left posterior leg and 4x10^6^ UM-SCC-74B cells (moderate to low CD44v6 expression) suspended in 200 µl 1:1 cell medium: matrigel were injected in the right posterior leg. Two weeks after tumor cell injections, experiments were performed. At the time of study, average animal weight was 18.8 ± 1.4 g.

Twelve mice bearing dual A431 and UM-SCC-74B xenografts received an intravenous injection via the tail vein with 100 µl ^125^I-A11(Iodogen) (234 kBq), and ^111^In-DTPA-A11 (500 kBq), in a total of 3 µg conjugate (1.5 μg of ^111^In-labeled and 1.5 μg ^125^I-labeled fragment). Another 13 mice received an intravenous injection via the tail vein with 100 µl ^125^I-H12(Iodogen) (173 kBq), and ^111^In-DTPA-H12 (500 kBq), in a total of 3 µg conjugate. At 4 h, 24 h and 48 h p.i. animals were euthanized with a mixture of ketamine and xylazine followed by heart puncture. Blood, tumors and organs of interest including salivary glands, thyroid (en bloc with larynx), tongue, heart, liver, kidneys, spleen, urinary bladder, colon, stomach, GI, skin, bone and muscle were collected, weighed and measured in a gamma well counter. The tail and the rest of the body were also measured. Three injection standards were measured for each group. Radioactivity uptake in the organ was calculated as percent of injected activity per gram of tissue (%ID/g). Uptake in thyroid was calculated as percent of injected activity per organ. Tumor-to-organ ratio was calculated as ratio activity/tumor divided by ratio activity/g_organ_.

#### Small animal PET-CT

Whole body PET-CT of ^124^I-H12(CAT) on the same animal model as above was performed under general anesthesia (isoflurane 1.0%-2.5% in 50% / 50% medical oxygen:air at 450 ml/min) at 48 h p.i.. Sedated xenograft with pre-injected tracer (7 μg, 5 MBq, in 110 μl PBS) was placed in the gantry of the small animal PET/CT scanner (TriumphTM Trimodality System, TriFoil Imaging, Inc., Northridge, CA, USA) and examined by a CT examination for 3 minutes (Field of View (FOV) = 8.0 cm) followed by whole body PET scan for 60 minutes in list mode. The breathing rate was monitored with a camera under controlled anesthesia. The animal was placed on the heated bed of the microPET scanner to prevent hypothermia and taped to prevent large movements during study. The PET data were reconstructed into a static image using an OSEM 3D algorithm (20 iterations). The CT raw files were reconstructed using Filter Back Projection (FBP). PET and CT DICOM files were fused and analyzed using PMOD v3.510 (PMOD Technologies Ltd, Zurich, Switzerland).

#### Digital autoradiography

Digital autoradiography of ^124^I-H12(CAT) on the A431 xenograft was performed *ex vivo* following the whole body PET-CT analysis. Tumor sections were placed on a phospholuminescence plate for approximately ten minutes. The plate was scanned in a Bio-Imaging Analyser BAS-1800II (FujiFilm Medical Systems), and the digital images were processed using Multi Gauge Image software (FujiFilm Medical Systems).

### Statistical analyses

Statistical analyses were performed using GraphPad Prism Version 6 for Windows (Graphpad Software Inc., La Jolla, CA, USA). If not otherwise stated, data are presented as mean ± standard deviation (SD). In blocking assays, differences between unblocked and blocked samples were assessed using student's t test and were considered significant if p<0.05. In biodistribution studies, significant differences between the groups over time were tested with one-way analysis of variance (ANOVA) followed by Newman-Keuls multiple comparison test. The differences in uptake between A431 and UM-SCC-74B xenografts were evaluated using one-way ANOVA with multiple comparison test. All tests described above were considered statistically significant if *P* < 0.05.

## SUPPLEMENTARY MATERIALS FIGURES



## Abbreviations

[^18^F]FDG: 2-deoxy-2-[^18^F]-fluoroglucose
ANOVA: Analysis of variance
BSA: Bovine serum albumin
CAT: Chloramine T,
CD44: *Cluster* of Differentiation 44
DICOM: Digital Imaging and Communications in Medicine
DOTA: 1,4,7,10-tetraazacyclododecane-1,4,7,10-tetraacetic acid
DTPA: Diethylenetriaminepentaacetic acid
CT: Computed tomography
EDTA: ethylenediaminetetraacetic acid
ELISA: Enzyme-Linked ImmunoSorbent Assay
Fab: Fragment antigen binding
Fc: Fragment crystallizable
FDG: Fludeoxyglucose (^18^F)
HNSCC: Head and neck squamous cell carcinoma
HRP: Horse radish peroxidase
IHC: Immunohistochemistry
ITLC: Instant thin layer chromatography
mAb: Monoclonal antibody
NBS: Sodium metabisulphite,
NRR: Negative regulatory region
OSEM: Ordered subset expectation maximization
PET: Positron emission tomography
PCR: Polymerase chain reaction
RU: Response units
SCC: Squamous cell carcinoma
scFv: Single chain fragment variable
SPECT: Single photon emission computed tomography
SPR: Surface plasmon resonance
VH: Variable heavy
VL: Variable light
