# Supplementary material for: Generation and evaluation of antibody agents for molecular imaging of CD44v6-expressing cancers
Source: Oncotarget. 2017 May 18;8(39):65152–70. doi: 10.18632/oncotarget.17996 (PMC5630320; doi:10.18632/oncotarget.17996)
Supplement: Supplementary file 1 [file oncotarget-08-65152-s001.pdf]

Generation and evaluation of antibody agents for molecular imaging of CD44v6-expressing cancers

SUPPLEMENTARY MATERIALS

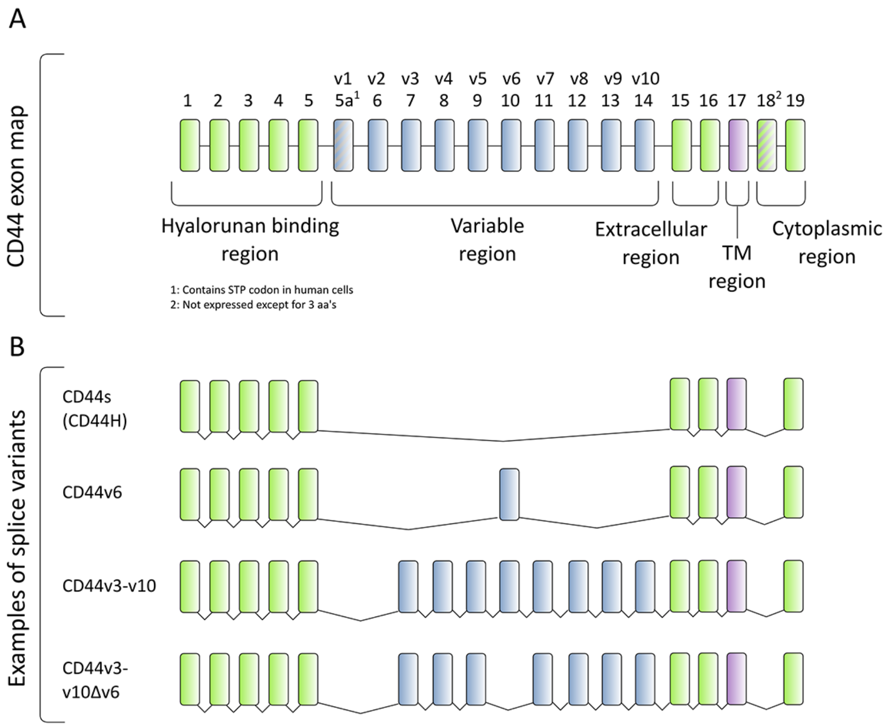

Supplementary Figure 1: (A) Gene map of CD44. **(B)** Standard CD44 (CD44s) does not contain variable exons. The exons v1-v10 are alternatively spliced [20].

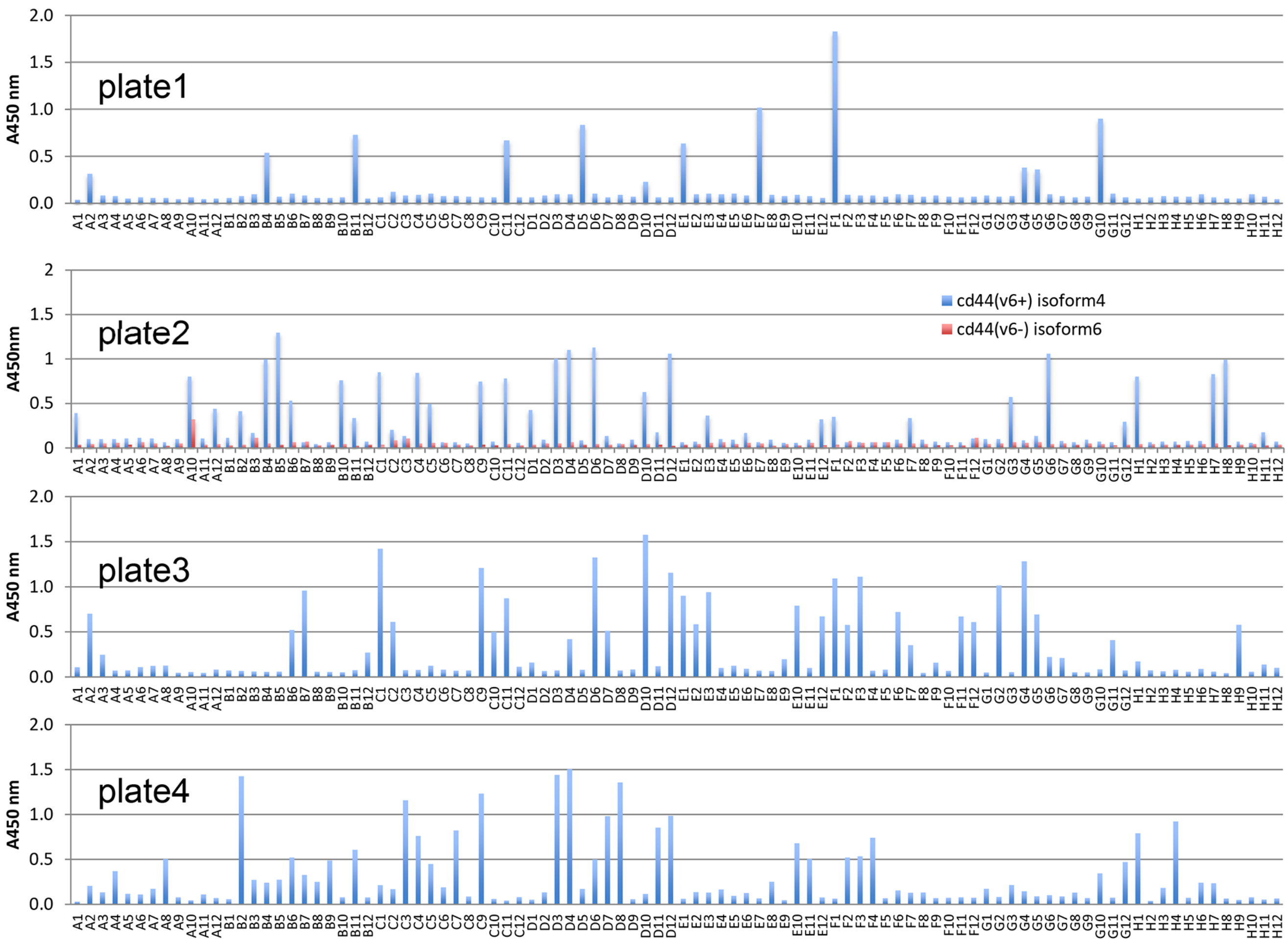

Supplementary Figure 2: Primary ELISA of 384 (4x96) scFv clones from 2 rounds of selection to CD44v6. No or very few (1/96) clones have cross reactivity to CD44(v6-) as demonstrated on plate2 above (red bars).
